# Prevalence and prognosis of left ventricular diastolic dysfunction in community hypertension patients

**DOI:** 10.1186/s12872-022-02709-3

**Published:** 2022-06-13

**Authors:** Dan Zhou, Mengqi Yan, Qi Cheng, Xiaoxuan Feng, Songtao Tang, Yingqing Feng

**Affiliations:** 1 Department of Cardiology, Guangdong Cardiovascular Institute, Guangdong Provincial People’s Hospital, Guangdong Academy of Medical Sciences, No. 106 Zhongshan 2nd Road, Yuexiu District, Guangzhou, 510080 People’s Republic of China; 2 Department of Internal Medicine, Community Health Center of Liaobu Community, Dongguan, 523411 China

**Keywords:** Left ventricular diastolic dysfunction, Major adverse cardiac events, Hypertension, Predictor

## Abstract

Left ventricular diastolic dysfunction (LVDD) is common in hypertension and is a predictor of increased cardiovascular risk, however the effect of LVDD, detected by new guideline, on major adverse cardiac events (MACE) is unknown in hypertensive patients without known cardiovascular disease. The present study aims to evaluate LVDD in a community hypertension cohort study and assess the effect of LVDD on MACE. we studied 283 asymptomatic nonischemic patients with hypertension who had baseline echocardiogram between 2012 and 2014. Patients were followed for MACE (myocardial infarction, coronary revascularization procedures, heart failure, stroke, all-cause mortality) with mean follow-up of 5.4 years. A Cox proportional hazards model was used to assess the association of LVDD with MACE. At baseline, 35 of the 283 hypertensions were diagnosed with LVDD (12.3%) and 25 patients were women (15.5%). Women had higher frequency of LVDD than men (8%). During follow-up, there were 26.6% patients occurring MACE in the LVDD group at baseline, 9.9% patients occurring MACE in the group with normal diastolic function. In multivariable Cox regression analyses, LVDD was a stronger predictor of MACE (HR: 2.5; 95% CI: 1.20 to 5.25; c- statistics 0.805) than E/e′ ratio (HR: 1.13; 95% CI: 1.04 to 1.22). LVDD was strongly associated with MACE in hypertension patients.

**Supplementary Information:**

The online version contains supplementary material available at 10.1186/s12872-022-02709-3.

## Introduction

Hypertension is a significant contributory factor to the development of major adverse cardiac events (MACE) [1–3]. In China, the individuals with hypertension have already exceeded 244 million [4]. Long-time chronic hypertension would cause cardiac remodeling due to the increased afterload. Left ventricular (LV) or left atrial remodeling is usually accompanied by systolic and diastolic dysfunction. Impaired LV relaxation is usually the initial performance of left ventricular diastolic dysfunction (LVDD) [5] caused by bad-controlled hypertension or its comorbidities, like type-2 diabetes mellitus, obesity or dyslipidemia. If there is no intervention for these comorbidities, some individuals would appear increased LV diastolic chamber stiffness and reduced passive ventricular, which increase LV filling pressure (LVFP).

The mechanisms, including increased afterload and myocardial fibrosis and inflammation [6], would cause LVDD in the community hypertensive individuals [7]. Echocardiography provides an easy, inexpensive and fast method to evaluate LVDD in community. LVDD is demonstrated to be a forerunner of heart failure, especially those heart failure with preserved ejection fraction (HFpEF) [8]. Previous studies using different diagnostic criteria have revealed the incident of LVDD between 20–58% in hypertension [9–11]. In Chinese community the prevalence of LVDD was 31.9%[12]. Previous studies assessed LVDD by the pulsed Doppler echocardiography or single parameter eʹ. LVDD detected by pulsed Doppler echocardiography was reported to be a predictor of adverse cardiovascular events independent of LV mass and ambulatory blood pressure in hypertension patients [13].

To provided standard evaluation of LVDD, the American Society of Echocardiography and the European Association of Cardiovascular Imaging guidelines recommended new process in 2016[14]. There are seldom studies in strict accordance with new guidelines to estimate LVDD in a hypertension cohort study, due to the lack of some parameters. The study aims to: (a) reveal the proportion of LVDD at baseline in a hypertension cohort study; and (b) determine the prognostic effect of LVDD on MACE.

## Methods

### Study participants

**Fig. 1:**
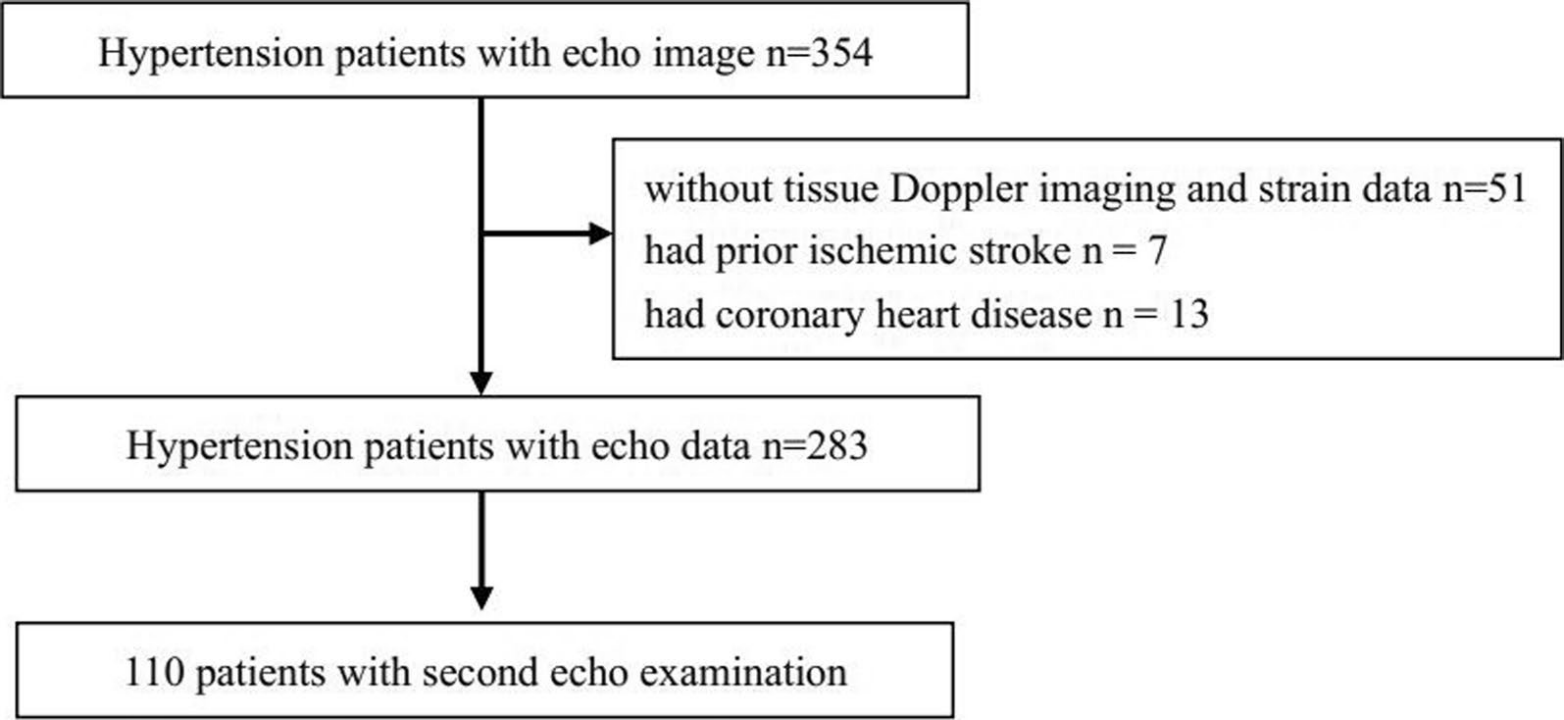
Study flow chart

We recruited hypertensive individuals from a cohort study conducted in the Community Health Center of Liaobu Community, Dongguan, Guangdong Province, China, as previously described [15]. The current study was approved by the Clinical Research Ethic Committee of Guangdong Provincial People's Hospital and the Liaobu County Health Department (No. GDREC2019343H). During the government-sponsored annual health examination in 2012 and 2014, we retrospectively included 354 hypertensive individuals underwent echocardiographic examination. The inclusion criteria are patients with hypertension defined by community physician or using anti-hypertensive drugs within past two weeks and sinus rhythm. The exclusion criteria are patients with significant valvular heart disease, cardiomyopathy, heart failure with reduced ejection fraction, atrial fibrillation, and with a poor imaging quality. Individuals who did not have tissue Doppler data or strain data (n = 51), had prior ischemic stroke (n = 7), and had coronary heart disease (n = 13) were excluded. Therefore, a total of 283 hypertensive individuals were included for the final analyses and 110 patients had second echo examination (Fig. 1). Written informed consent was obtained before enrollment.

### Clinical variables

Clinical characteristics (age, sex, smoking, medical history, antihypertensive medication history) were collected using standardized questionnaire by trained community staffs. Weight and height were measured in participants wearing light clothing and standing with no shoes. Body mass index (BMI) was calculated as BMI = weight/height (kg/m^2^) [16]. Body surface area (BSA) was calculated as BSA = (Weight ^0.425^ × Height ^0.725^) × 0.007184. Blood samples were taken after at least 8 h fasting. The blood samples were used to assess fasting plasma glucose, uric acid, low-density lipoprotein cholesterol and serum creatinine. Estimated glomerular filtration rate (eGFR) was calculated by using the Modification of Diet in Renal Disease formula, [17]and eGFR < 60 ml/min/1.73 m^2^ was defined as chronic kidney disease (CKD).

### Blood pressure (BP) measurement

According to the China guideline's recommendation[18], BP was taken twice in the sitting position after 5 min rest with 1–2 min interval using Omron HEM-7051 device (Omron HealthCare, Guangzhou, China). The average value of two BP readings was recorded. If the first two BP readings differed by > 5 mm Hg, the third measurement was required, and the mean value of three readings was used. Heart rate was obtained using Omron HEM-7051 device.

### Echocardiographic examination

According to the guideline's recommendation [14], we use a Vivid S6 Ultrasound instrument (GE Ving-Med, Guangzhou, China) interfaced with a M4S-RS Probe with 2.5- to 3.5-MHz phased-array to obtain imaging and stored in DICOME format. Left atrial volume (LAV), LV end-diastolic volume (LVEDV) and LV end-systolic volume (LVESV) were assessed using the modified biplane Simpson's rule from the 4-chamber view and was indexed to BSA. LV end-diastolic diameter (LVEDD), LV posterior wall (LVPW) and interventricular septum thickness (IVS) in diastole were used to calculate left ventricular mass (LVM) and were indexed to BSA. LVM was calculated as LVM = 0.8* 1.04 *[(IVS + LVEDD + LVPW)^3^—LVEDD^3^] + 0.6 g. LVM index (LVMI) ≥ 115 g/m^2^ in men and ≥ 95 g/m^2^ in women were defined as LVH [19]. Mitral inflow velocity (peak E- and A-wave) and peak early systolic tissue velocity (s′) and peak early diastolic tissue velocity (e′) were measured from the 4-chamber view. According to the guideline recommendation [14], septal e′ velocity, lateral e′ velocity, average E/e′ ratio, LAV index and tricuspid regurgitation velocity (TRV) were used to define LVDD (Additional file 1: Fig. 1) and elevated average E/e′ ratio was defined as an increased LVFP. We found only 69 patients had TRV for analysis, and 33 had TRV > 2.8 m/s, 36 had 2.0 m/s < TRV ≤ 2.8 m/s (Additional file 1: Table 2). When defined LVDD, others who had no Continuous Doppler Spectrum from Tricuspid regurgitation to analysis were recognized less than 2.8 m/s (Additional file 1: Table 2). we suggested a cut-point of 16% in absolute values and a value below 16% is abnormal global longitudinal strain (GLS) [20]. Myocardial strain parameter GLS was used by standard methodologies for speckle tracking (Echo PAC201; GE Ving-Med). GLS was calculated averaging the negative peak of longitudinal strain from 18 ventricular segments from the apical 4-chamber, 2-chamber, 3-chamber views. [21].

### Outcome

The primary outcome was MACE (myocardial infarction, coronary revascularization procedures, heart failure, stroke, all-cause mortality). Outcomes were collected from Dongguan Medical Insurance Bureau. This captures all admission data in all public hospitals when submitting medical expense. Patients were censored at the time of outcome or at the end of follow-up (December 31, 2018).

### Interobserver variability

To test the reproducibility of echocardiographic measurements, the key parameters, including Septal e′ velocity, Lateral e′ velocity, average E/e′, LAV index and TRV, were remeasured in 30 randomly selected subjects from the hypertensive patients. Interobserver variability was assessed between two investigators (Z.D. and Y.M.Q.).

Reliability was assessed using an intraclass correlation co-efficient (ICC) (Additional file 1: Table 3).

### Statistical analysis

Distribution normality was measured by Shapiro–Wilk normality test. Continuous variables without normal distribution should be shown as median and interquartile range (IQR). Continuous variables with normal distribution are summarized by mean ± standard deviation. Categorical variables are summarized by the frequency (%). Continuous variables were tested for normality using the Kolmogorov–Smirnov test. The differences between groups were tested by t-test, the Mann–Whitney, the chi-square test or Fisher exact test. First, we use univariable models to select variables (p < 0.05 or conventional risk factors were included in multivariable models) and the multivariable Cox proportional hazards models were used to determine the risk factors associated with MACE. Two models (a clinical and an echocardiographic model) were created to avoid overfitting. The first step consisted of fitting a multivariable model of age, female, diastolic BP. Then, E/e′ ratio was included in the second step. Finally, LVDD was included in the third step. The change in overall log-likelihood ratio was used to assess the increase in predictive power. We used C statistic to evaluate model by logistic regression analyses and receiver operating characteristic curve (ROC). Survival was estimated by the Kaplan–Meier method, and any difference in survival were evaluated with a stratified log-rank test. Statistical analysis was performed using SPSS, version 25, and statistical significance was defined by p < 0.05 (2-tailed).

## Results

### Baseline characteristics

The 283 individuals (mean age 63 years) included 161 women, mostly had controlled blood pressure (mean SBP, 138 mmHg), with comorbid diseases (type 2 Diabetes mellitus, 15.1%, CKD, 14.8%). All were taking antihypertensive medications. 90.7% of individuals use angiotensin receptor blocker or calcium channel blockers to lower blood pressure. The mean LVMI was 92 g/m^2^. Although LVEF (mean EF, 68%) was normal, GLS (mean, 15.6%) was abnormal in most patients. Although the LAVI (mean 26 ml/m^2^) was normal, Septal e′ velocity (mean, 6.7 cm/s), Lateral e′ velocity (mean, 8.8 cm/s) and average E/e′ ratio (mean, 9.9) was abnormal in most patients (Table 1). Functional parameters showed that 74.2% patients had lower Septal e′ (< 7 cm/s), 76.6% had lower Lateral e′ (< 10 cm/s), 7.7% had higher E/e′ ratio (> 14), 14.5% had higher LAVI (> 34 ml/m^2^), 12.3% had LVDD, 50.2% had abnormal GLS. Morphological abnormalities showed 35% of patients had LVH.

**Table 1:** Baseline characteristics

Variables	N = 283	Men (n = 122)	Women (n = 161)	p-value
Age (years)	63 ± 11	61 ± 13	64 ± 10	0.09
SBP (mm Hg)	138 ± 16	138 ± 18	138 ± 16	0.823
DBP (mm Hg)	82 ± 9	83 ± 10	82 ± 8	0.429
HR (beat per minute)	71 ± 10	71 ± 11	72 ± 11	0.725
BMI (kg/m2)	24 ± 3.6	24.4 ± 3	24.7 ± 4	0.510
Smoking, *n* (%)	63 (22.2%)	57 (46.8%)	6 (3.7%)	< 0.001
Diabetes mellitus, *n* (%)	43 (15.1%)	16 (13.9%)	27 (16.7%)	0.318
CKD, *n* (%)	42 (14.8%)	23 (19.1%)	19 (11.8%)	0.091
FPG (mmol/L)	5.3 ± 1.6	5.2 ± 1	5.4 ± 1	0.310
LDL cholesterol (mg/dl)	102 ± 31	99 ± 33	101 ± 29	0.549
Creatinine (µmol/L)	78 ± 24	89 ± 24	70 ± 20	< 0.001
eGFR (ml/min/1.73 m2)	81 ± 20	80 ± 21	82 ± 19	0.523
Uric acid (µmol/L)	410 ± 115	449 ± 117	383 ± 108	< 0.001
HGB (g/L)	13.1 ± 13	138 ± 13	126 ± 12	< 0.001
ACEI, *n* (%)	36 (12.7%)	18 (14.7%)	18 (11.1%)	0.284
ARB, *n* (%)	139 (49.1%)	60 (49.5%)	79 (49.0%)	0.537
CCB, *n* (%)	118 (41.6%)	51 (42.2%)	67 (41.6%)	0.531
Diuretic, *n* (%)	9 (3.1%)	7 (6.4%)	4 (2.4%)	0.127
Betablocker, *n* (%)	29 (10.2%)	16 (13.8%)	13 (8.0%)	0.105
LVEDV index (ml/m^2^)	56 ± 12	55 ± 12	56 ± 11	0.669
LVESV index (ml/m^2^)	17 ± 3	17 ± 6	17 ± 6	0.686
LVMI (g/m^2^)	92 ± 20	92.3 ± 20	92.4 ± 20	0.960
LVH, n (%)	99 (35%)	26 (21.4%)	73 (45.3%)	< 0.001
Septal S′ velocity (cm/s)	7.2 ± 1.4	7.4 ± 1.4	7.1 ± 1.4	0.247
LVEF (%)	68 ± 7	69 ± 8	68 ± 7	0.597
Septal e′ velocity (cm/s)	6.7 ± 1.9	6.8 ± 1.9	6.5 ± 1.8	0.179
Septal e′ velocity < 7 cm/s, *n* (%)	210 (74.2%)	89 (73.2%)	121 (75%)	0.426
Lateral e′ velocity (cm/s)	8.8 ± 2.7	9.1 ± 2.5	8.6 ± 2.7	0.136
Lateral e′ velocity < 10 cm/s, *n* (%)	217 (76.6%)	85 (69.6%)	132 (81.9%)	0.023
Average E/e′ ratio	9.9 ± 3.0	9.7 ± 3	10 ± 3	0.591
Average E/e′ ratio > 14, *n* (%)	22 (7.7%)	8 (6.5%)	14 (8.7%)	0.303
LAVI (ml/m^2^)	26 ± 8.6	25 ± 8	27 ± 8	0.089
LAVI > 34 ml/m^2^, *n* (%)	41 (14.5%)	12 (9.8%)	29 (18.2%)	0.041
LVDD, n (%)	35 (12.3%)	10 (8%)	25 (15.5%)	0.049
GLS (%)	15.6 ± 3.6	16% ± 3	15.5% ± 3	0.290
GLS < 16%, n (%)	142 (50.2%)	60 (49.2%)	82 (50.9%)	0.279

Data showed mean ± SD or number (percentage)

SBP: systolic blood pressure; DBP: diastolic blood pressure; HR: Heart rate; BMI: Body mass index; CKD: chronic kidney disease; FPG: fast plasma glucose; LDL: low density Lipoprotein; eGFR: estimate glomerular filtration rate; HGB: Hemoglobin; ACEI: angiotensin converting enzyme inhibitors; ARB: Angiotensin Receptor Blocker; CCB: Calcium Channel Blockers; LVEDV: left ventricular end-diastolic volume; LVESV: left ventricular end-systolic volume; LVMI: Left ventricular mass index; LVH: Left ventricular hypertrophy; LVEF: Left ventricular ejection fraction; LAVI: Left atrial volume index; LVDD: Left ventricular diastolic dysfunction; GLS: global longitudinal strain;

### LV systolic and diastolic function in women and men

In Table 1, diastolic function differed by sex. Women had significant higher proportion of decreased Lateral e′ velocity (81.9%), increased LAVI (18.2%) and higher prevalence of LVDD (15.5%) than men (8%) (all p < 0.05). Septal e′ velocity and Average E/e′ ratio showed no difference by sex. Systolic function indicators like Septal S′ velocity, LVEF, and GLS, showed no difference among women and men. Women had significant higher prevalence of LVH (45.3%) than men (21.4%) (p < 0.001), although the mean LVMI showed no difference by sex.

### Outcomes

During follow-up (mean 5.4 years), 45 patients (15.9%) suffered MACE (28 deaths and hospital admissions caused by MACE, including 6 admissions with heart failure or acute myocardial infarction, and 11 with stroke).

### Association between baseline study parameters and MACE

Table 2 compares the clinical and echo parameters in hypertensive patients with and without MACE. MACE was significantly associated with older, lower DBP, higher prevalence of CKD, higher use of ACEI, higher LVMI, higher prevalence of LVH, greater impairment of diastolic function (lower septal e′ velocity, lower Lateral e′ velocity, higher average E/e′ ratio, elevated LAVI) and present of LVDD. In the echo parameters, the prevalence of LVDD (26.6%) was significant higher in patients with MACE. MACE showed no significant associated with systolic function in univariable analysis. Septal S′ velocity, LVEF, and GLS showed no difference between the group.

**Table 2:** Univariable Cox regression analysis for the association of MACE

Variables	MACE (n = 45)	NON-MACE (n = 238)	HR (95%CI)	*P* value
Age (years)	72 ± 9	61 ± 11	1.09 (1.05–1.12)	< 0.001
SBP (mm Hg)	139 ± 17	138 ± 17	1.01 (0.99–1.02)	0.166
DBP (mm Hg)	78 ± 10	83 ± 9	0.93 (0.90–0.97)	< 0.001
HR (beat per minute)	72 ± 10	71 ± 11	1.00 (0.97–1.03)	0.361
BMI (kg/m^2^)	23.8 ± 3.9	24.8 ± 3.5	0.95 (0.87–1.03)	0.254
Women (%)	24 (53.3%)	137 (57.6%)	1.51 (0.68–3.34)	0.308
Smoking, (%)	10 (23.8%)	53 (22.2%)	1.61 (0.64–4.01)	0.305
Diabetes mellitus, *n* (%)	8 (17.7%)	35 (14.7%)	1.17 (0.49–2.78)	0.721
CKD, *n* (%)	14 (31%)	28 (11.7%)	3.33 (1.72–6.46)	< 0.001
FPG (mmol/L)	5.4 ± 2.2	5.3 ± 1.4	1.05 (0.89–1.23)	0.561
LDL cholesterol (mg/dl)	94 ± 28	102 ± 31	0.99 (0.97–1.00)	0.115
Creatinine (µmol/L)	88 ± 32	77 ± 21	0.99 (0.99–1.00)	0.417
eGFR (ml/min/1.73 m^2^)	71 ± 23	83 ± 19	0.99 (0.97–1.02)	0.914
Uric acid (µmol/L)	406 ± 115	410 ± 116	1.00 (0.99–1.00)	0.816
HGB (g/L)	128 ± 14	132 ± 13	0.98 (0.96–1.00)	0.087
ACEI, *n* (%)	11 (25.6%)	25 (10.5%)	2.57 (1.26–5.24)	0.040
ARB, *n* (%)	18 (40%)	121 (50.8%)	1.18 (0.62–2.25)	0.599
CCB, *n* (%)	22 (48.8%)	96 (40.3%)	0.67 (0.34–1.31)	0.250
Diuretic, *n* (%)	1 (2.2%)	8 (3.3%)	2.31 (0.30–17.90)	0.421
Betablocker, *n* (%)	8 (18.6%)	21 (8.8%)	0.48 (0.20–1.11)	0.108
LVEDV index (ml/m^2^)	59 ± 13	55 ± 12	1.03 (0.95–1.13)	0.417
LVESV index (ml/m^2^)	19 ± 6	17 ± 6	0.88 (0.67–1.14)	0.342
LVMI (g/m^2^)	101 ± 24	90 ± 19	1.02 (1.00–1.03)	0.002
LVH, n (%)	24 (53.5%)	75 (31.5%)	1.94 (1.07–3.50)	0.028
Septal S′ velocity (cm/s)	6.6 ± 1.4	7.3 ± 1.4	0.88 (0.67–1.15)	0.370
LVEF (%)	67 ± 7.7	69 ± 7.3	0.90 (0.76–1.06)	0.222
LAVI (ml/m^2^)	30 ± 12	26 ± 7	1.06 (1.03–1.09)	< 0.001
Septal e′ velocity (cm/s)	5.5 ± 1.6	6.9 ± 1.9	0.63 (0.50–0.78)	< 0.001
Lateral e′ velocity (cm/s)	7.6 ± 2.4	9.0 ± 2.7	0.81 (0.71–0.93)	0.003
Average E/e′ ratio	12.1 ± 3.9	9.5 ± 2.6	1.23 (1.14–1.34)	< 0.001
LVDD, n (%)	12 (26.6%)	23 (9.9%)	3.09 (1.54–6.22)	0.001
GLS (%)	14.9 ± 3.3	15.7 ± 3.7	1.05 (0.97–1.14)	0.192

Data showed mean ± SD or number (percentage)

SBP: systolic blood pressure; DBP: diastolic blood pressure; HR: Heart rate; BMI: Body mass index; CKD: chronic kidney disease; FPG: fast plasma glucose; LDL: low density Lipoprotein; eGFR: estimate glomerular filtration rate; HGB: Hemoglobin; ACEI: angiotensin converting enzyme inhibitors; ARB: Angiotensin Receptor Blocker; CCB: Calcium Channel Blockers; LVEDV: left ventricular end-diastolic volume; LVESV: left ventricular end-systolic volume; LVMI: Left ventricular mass index; LVH: Left ventricular hypertrophy; LVEF: Left ventricular ejection fraction; LAVI: Left atrial volume index; LVDD: Left ventricular diastolic dysfunction; GLS: global longitudinal strain; HR: hazard risk

### Incremental value of LVDD

**Fig. 2:**
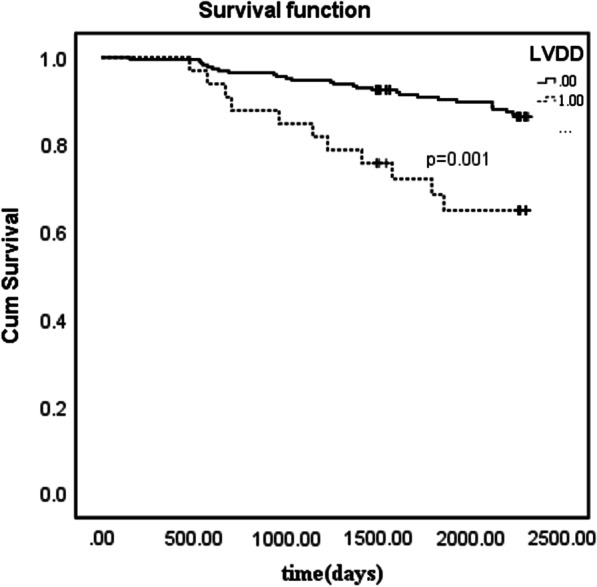
Kaplan–Meier analysis of hypertension patients, segregated by left ventricular diastolic dysfunction at baseline echocardiography

In the multivariable regression analyses (Table 3), age, average E/e′ ratio, and LVDD showed significant associated with MACE. In both clinical and echo models, LVDD all showed independently associated with MACE. In sequential Cox models, the model based on clinical variables was significantly improved by the addition of E/e′ ratio, and furthermore improved by adding LVDD in Table 4. LVDD independently predicted MACE (HR: 2.5; 95% CI 1.2–5.2; p = 0.032) in a model including age, sex, DBP, and E/e′ ratio (c- statistics 0.805). Survival curve was compared by Kaplan–Meier analysis according to the present of LVDD (Fig. 2). The patients with LVDD had higher risk of MACE (P < 0.001).

**Table 3:** Characteristics independently associated with MACE (multivariable Cox regression)

	Clinical Model Chi-Square, 31.9 C Statistic, 0.803 HR (95% CI)	p-value	Echo Model Chi-Square, 28.5 C Statistic, 0.736 HR (95% CI)	p-value
Age (years)	1.09 (1.05–1.12)	< 0.001		
Women	1.14 (0.57–2.27)	0.405		
DBP (mm Hg)	0.97 (0.93–1.00)	0.108		
CKD, n (%)	1.59 (0.74–3.40)	0.272		
ACEI, *n* (%)	2.18 (0.99–4.76)	0.115		
LVMI (g/m^2^)			0.99 (0.97–1.02)	0.828
LVH			1.29 (0.46–3.65)	0.621
Septal e′ velocity (cm/s)			0.75 (0.58–3.96)	0.067
Lateral e′ velocity (cm/s)			0.86 (0.74–4.06)	0.051
Average E/e′ ratio			1.16 (1.05–1.29)	0.002
LAVI (ml/m^2^)			1.03 (0.99–1.07)	0.102
LVDD	2.49 (1.19–5.20)	0.014	2.63 (1.24–5.57)	0.012

DBP: diastolic blood pressure; CKD: chronic kidney disease; ACEI: angiotensin converting enzyme inhibitors; LVMI: Left ventricular mass index; LVH: Left ventricular hypertrophy; LAVI: Left atrial volume index; LVDD: Left ventricular diastolic dysfunction; HR: hazard risk

**Table 4:** Incremental value of LVDD over clinical parameters and E/e′ ratio as a correlate of MACE

Variable	Model 1 (Clinical) Chi-Square, 33.7 HR (95% CI), p Value	Model 2 (Clinical + E/e′) Chi-Square, 37.0 HR (95% CI), p Value	Model 3 (Clinical + E/e′ + LVDD) Chi-Square, 43.1 HR (95% CI), p Value
C-statistics	0.775	0.793	0.805
Age (per 1 year increase)	1.09 (1.05–1.12) p < 0.001	1.09 (1.05–1.12) p < 0.001	1.07 (1.04–1.11) p < 0.001
Women	1.26 (0.69–2.29) p = 0.436	1.54 (0.82–2.91) p = 0.176	1.48 (0.77–2.85) p = 0.230
DBP (per 1 mm Hg increase)	0.98 (0.94–1.01) p = 0.244	0.97 (0.93–1.00) p = 0.115	0.96 (0.93–1.00) p = 0.084
Average E/e′ ratio (per 1 unite increase)		1.14 (1.05–1.25) P = 0.002	1.13 (1.04–1.22) P = 0.002
LVDD			2.50 (1.20–5.25) P = 0.032

DBP: diastolic blood pressure; LVDD: Left ventricular diastolic dysfunction; HR: hazard risk

### Longitudinal assessment of LVDD

After a median follow-up of 1.5 years, 110 patients had second echo data evaluable for diastolic function. LVDD was diagnosed in 33 patients (30%) at follow up (Additional file 1: Fig. 2). Individual diastolic function parameter analyses showed that the septal eʹ and lateral eʹ were worsen at follow-up than at baseline, although E/e′ ratio and LAVI did not significant increase (Additional file 1: Table 1).

## Discussion

The authors found the prevalence of LVDD, defined by new guidelines in 2016, was 12.3% in the baseline [14]. Women had worse LV diastolic function and higher proportion of LVDD in the baseline. Above all, LVDD was associated with MACE in hypertension patients, independent of separated diastolic functional parameters like e′, and LAV index, LVH and traditional clinical parameters.

The results showed a 12.3% cumulative incidence of LVDD at baseline. The proportion is in the lower range of previous reports [9–11] and may represent actual population discrepancy from other studies. However, it also may be due to the stricter definitions of LVDD defined by new guidelines in 2016 and the patients were recruited from community in our study, potentially excluding patients with heavier disease. If we defined LVDD with single parameters, the results were nearly consistent with previous report [9]. We also found the proportion of single abnormal e′ velocity was high to 70%.

We confirm that in hypertensive patients without cardiovascular disease, septal e′ velocity and lateral e′ velocity was lower in women than men in trend, in the contrary, E/e′ ratio and LAVI was higher [22]. The report from Okura showed that in patients younger than 50 years, e′ velocity was higher in women; while in those older than 70 years, e′ velocity was lower in women [23]. The results from Cai showed septal e′ velocity was lower in women in those 55 years or older [24]. Chronic increased left ventricular end-diastolic pressure leads to increasement of LAVI, an important index of LVDD. Research results are inconsistent regarding the gender difference in LAVI. One study reported that women was positively associated with LAVI by cardiac magnetic resonance [25]. D'Andrea and colleagues reported that in healthy individuals, LAVI was correlated to age but not sex, simultaneously, another community hypertension study proved the association did not significantly differ by sex [24, 26]. Inconsistent with prior reports, the current study demonstrated that women had increased LAVI compared with men. The overall findings suggest that in hypertensive patients without cardiovascular disease, women had more frequent of LVDD. A possible explanation for impairment of LV relaxation in elderly women may be the lack of adequate estrogen after menopause [27]. The reason for this rather contradictory result is still not completely clear. Indeed, prior study has demonstrated a beneficial effect of estrogen replacement therapy on improvement of LV diastolic function [28].

Impaired LV early diastolic relaxation identifies hypertensive patients at increased cardiovascular risk independently of LV mass and ambulatory BP [13]. Prior report demonstrated that e′ velocity was a significant predictor of fatal and nonfatal cardiovascular events in a general population and the diastolic dysfunction group characterized by elevated LVFP, as indexed by E/e′ ratio, had higher cardiovascular events [29]. LVDD, as indexed by e′ < 5.8 cm/s, is independently related to increased risk for cardiac events or cardiovascular hospitalization in patients with known or suspected cardiovascular disease [30]. Prior studies demonstrated LVDD, as indexed by pulsed Doppler echocardiography or e′, is independently associated with cardiac events. Many studies are inconsistent in the definition of LVDD, although the new guidelines have recommended. The reason may be that the indicators are difficult to obtain completely or the deficiency of tissue Doppler equipment in earlier time.

Although e′ is a maker of left ventricular relaxation, evaluation of diastolic function is recommended to rely on combined parameters (septal e′, lateral e′, E/e′ ratio, LAVI, TRV). Our study confirmed LVDD, as indexed by guideline in 2016[14], was an independent predictor for MACE and had incremental prognostic effect than E/e′ ratio. The mean age of 62 years and a mean follow-up of 5.4 years may illustrate the high mortality. Eventually, both age and LVDD predicted MACE independently. If the effect on MACE from LVDD was due to higher age, we would expect only age to remain an independent predictor and not both age and LVDD remained significant predictors.

In the follow-up echo, septal and lateral e′ velocity was significantly lower than baseline, although the patients were assigned anti-hypertensive treatment by community physicians. LAVI and E/e′ ratio showed no difference. We hypothesize that value of left atrial size and E/e′ ratio is response to prolonged stress effects and may have changed slowly in 1.5 years. Prior study [31] showed that it may require at least 3 years of aggressive antihypertensive treatment for maximum improvement in LV diastolic filling patterns, which may too short time to show difference in the current study.

Our data should be interpreted in the background of some limitations. First, the sample of the study was small. The group comprised asymptomatic patients who had a relatively mild clinical condition with short follow-up time. More patients with long follow-up time are necessary to confirm the independent association between them. In order to explore the Longitudinal change of LVDD, we showed the second echo of 110 patients. Due to the lost to follow up is more than 50%. The results of Longitudinal change of LVDD maybe biased. In the future, we would explore the relationship between progress or reversal of LVDD and MACE with longer follow-up time. Second, variation of diastolic function over time can’t be illustrate well. More patients are necessary to confirm the association in the variation of LVDD. Thirdly, the baseline blood pressure was monitored in one time, it can’t reflect the conditions during the cohort study. We did not have the blood pressure data in the follow-up.

## Conclusions

The prevalence of LVDD in the present study was 12.3% in the baseline. Women had worse LV diastolic function and higher proportion of LVDD in the baseline. The new guideline was more stricter and specificity than previous criterion. LVDD was an independent predictor of MACE in hypertension patients, independent of separated diastolic function, LVH and clinical parameters. Strict LVDD definition would help distinguishing patients with greater risk. In community hypertensive individuals, it’s necessary to perform the evaluation of diastolic function. In further studies, we aim to explore the factors of reversing LVDD and the association with cardiac events.

## Supplementary Information

**Additional file 1**. **Table S1.** Diastolic parameters in patients with available measurements at both baseline and follow-up echocardiography. **Table S2.** Tricuspid regurgitation velocity in patients with MACE or not. **Table S3.** The intraclass correlation coefficients of interobserver reproducibility. **Figure S1.** Algorithm for diagnosis of LV diastolic dysfunction in subjects with normal LVEF. **Figure S2.** Distribution of LVDD in hypertensive patients. Boxes show hypertensive patients segregated by LVDD from baseline to follow-up.

## Data Availability

Some or all data, models, or code generated or used during the study are available from the corresponding author by request.
