# Supplementary material for: Prevalence and prognosis of left ventricular diastolic dysfunction in community hypertension patients
Source: BMC Cardiovasc Disord. 2022 Jun 13;22:265. doi: 10.1186/s12872-022-02709-3 (PMC9195252; doi:10.1186/s12872-022-02709-3)
Supplement: Supplementary file 1 — Additional file 1. Table S1. Diastolic parameters in patients with available measurements at both baseline and follow-up echocardiography. Table S2. Tricuspid regurgitation velocity in patients with MACE or not. Table S3. The intraclass correlation coefficients of interobserver reproducibility. Figure S1. Algorithm for diagnosis of LV diastolic dysfunction in subjects with normal LVEF. Figure S2. Distribution of LVDD in hypertensive patients. Boxes show hypertensive patients segregated by LVDD from baseline to follow-up. [file 12872_2022_2709_MOESM1_ESM.docx]

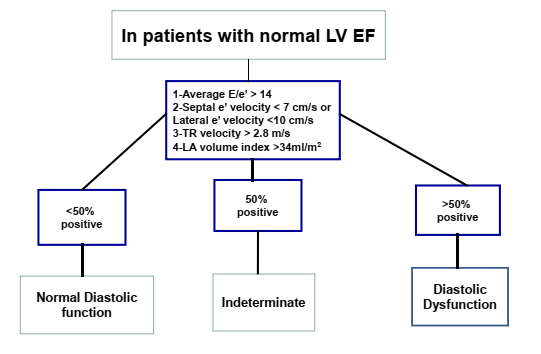


Supplement Figure 1 Algorithm for diagnosis of LV diastolic dysfunction in subjects with normal LVEF(1).

1. Nagueh SF, Smiseth OA, Appleton CP, Byrd BF, 3rd, Dokainish H, Edvardsen T, et al. Recommendations for the Evaluation of Left Ventricular Diastolic Function by Echocardiography: An Update from the American Society of Echocardiography and the European Association of Cardiovascular Imaging. European heart journal Cardiovascular Imaging. 2016;17(12):1321-60.


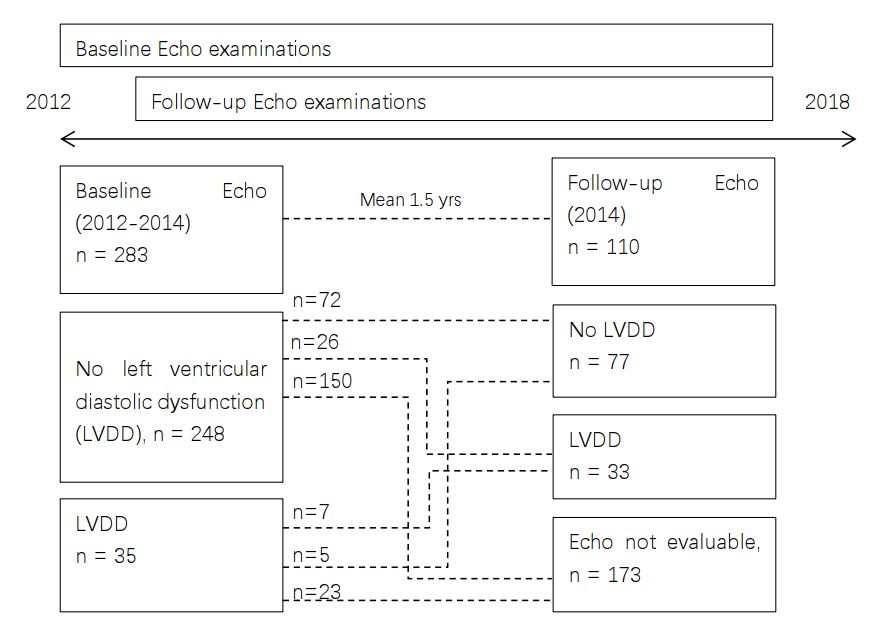


Supplement Figure 2 Distribution of LVDD in hypertensive patients.

Boxes show hypertensive patients segregated by LVDD from baseline to follow-up.

**Supplement Table 1 Diastolic parameters in patients with available measurements at both baseline and follow-up echocardiography**

|  | Baseline  Echocardiography | Follow-Up  Echocardiography | p Value |
| --- | --- | --- | --- |
| Septal e’ velocity (cm/s) | 6.8±2.0 | 5.8±1.8 | <0.001 |
| Lateral e’ velocity (cm/s) | 9.2±2.6 | 8.2±2.6 | <0.001 |
| Average E/e’ ratio | 9.4±2.6 | 9.8±3.1 | 0.117 |
| LAVI (ml/m2) | 26±7.5 | 28±10 | 0.098 |

LAVI: Left atrial volume index.

**Supplement Table 2 Tricuspid regurgitation velocity * in patients with MACE or not**

| Variables | MACE(n=20) | NON-MACE(n=49) | p-value |
| --- | --- | --- | --- |
| Tricuspid regurgitation velocity(m/s) | 2.65 | 2.60 | 0.554 |

* Only 69 patients had TR velocity for analysis.

Supplement Table3 The intraclass correlation coefficients of interobserver reproducibility.

|  | ICC | P value |
| --- | --- | --- |
| Septal e’ velocity | 0.90 | <0.001 |
| Lateral e’ velocity | 0.91 | <0.001 |
| average E/e’ ratio | 0.85 | <0.001 |
| LAV index | 0.84 | <0.001 |
| TRV | 0.87 | <0.001 |

LAV: Left atrial volume, TRV: tricuspid regurgitation velocity
